# Warming in combination with increased precipitation mediate the sexual and clonal reproduction in the desert steppe dominant species *Stipa breviflora*

**DOI:** 10.1186/s12870-023-04439-w

**Published:** 2023-10-09

**Authors:** Saixiyala, Lingling Chen, Fengyan Yi, Xiao Qiu, Hailian Sun, Hongxia Cao, Taogetao Baoyin, Xuehua Ye, Zhenying Huang

**Affiliations:** 1 Ministry of Education Key Laboratory of Ecology and Resource Use of the Mongolian Plateau and Inner Mongolia Key Laboratory of Grassland Ecology, School of Ecology and Environment, Inner Mongolia University, Hohhot, 010021 China; 2 Inner Mongolia Academy of Agricultural and Animal Husbandry Sciences, Hohhot, 010030 China; 3 Suzhou Vocational Technical College, Suzhou, 234099 China; 4 State Key Laboratory of Vegetation and Environmental Change, Institute of Botany, Chinese Academy of Sciences, No.20 Nanxincun, Xiangshan, Beijing, 100093 China

**Keywords:** Climate change, Reproductive ramet, Tillerous clonal plant, Trade-off, Vegetative ramet

## Abstract

**Background:**

Clonal plants can successfully adapt to various ecosystems. A trade-off between sexual and clonal reproduction is generally assumed in clonal plants, which may be influenced both by the characteristics of the plant itself and environmental conditions. Currently, it is unclear how climate change, and specifically warming and increased precipitation, might affect sexual and clonal reproduction in clonal plants. Therefore, this study aimed to investigate both the sexual and clonal reproduction responses of *Stipa breviflora* to warming and increased precipitation. A controlled experiment was conducted by inducing increases in precipitation (ambient condition, 25% and 50% increases) and warming (ambient temperature, 1.5 °C and 3.0 °C increases).

**Results:**

Warming significantly influenced both the ratio of reproductive ramet shoot biomass to total shoot biomass, and the ratio of reproductive ramet number to total ramet number. Additionally, the ratio of reproductive ramet shoot biomass to total shoot biomass was also significantly affected by increased precipitation. Increased precipitation benefited sexual reproduction, while effects of warming on reproductive and/or vegetative ramets varied from negative to positive depending on precipitation conditions. There was no relationship between the number or shoot biomass of reproductive ramets and vegetative ramets. Reproductive ramets displayed greater sensitivity to climate change than vegetative ramets.

**Conclusions:**

The findings of our study suggest that there was no trade-off between sexual and clonal reproduction in *S. breviflora*. The combined impact of warming and increased precipitation promoted sexual reproduction but did not inhibit clonal reproduction. Clonal plants with the capacity for both sexual and clonal reproduction, may cope with climate change well via clonal reproduction, ensuring their survival.

**Supplementary Information:**

The online version contains supplementary material available at 10.1186/s12870-023-04439-w.

## Background

Clonal plants can successfully adapt to new environments, allowing them to dominate many ecosystems [27, 52] owing to traits such as clonal plasticity, which enables the production of different phenotypes in different environments [59, 61], clonal integration, facilitating translocation and sharing of resources and/or signals between ramets connected by clonal organs [60, 70]; clonal foraging, leading to the allocation of more roots, shoots, and/or whole ramets in high-resource patches [9, 10, 72], and trade-offs between different clonal growth forms [73, 76]. Most clonal plants have the capacity for both sexual and clonal reproduction [22, 27, 28, 41]. Sexual reproduction via seeds enables long-distance seed dispersal, reduces local intraspecific competition, and ensures genetic diversity, whereas clonal reproduction mainly contributes to local population growth and high resilience to herbivory, drought and other stresses [18, 56, 80]. Sexual and clonal reproduction are thought to compete for limited resources within a plant, leading to potential trade-offs between sexual and clonal reproduction. Indeed, such trade-offs have been demonstrated in various species, such as *Ligularia virgaurea* [65], *Spartina alterniflora* [68], *Sagittaria latifolia* [56], *Carex brevicuspis* [13], and *Duchesnea indica* [67].

The trade-offs between sexual and clonal reproduction caused by environmental factors may be more complex than initially believed. Loehle [35] predicted that clonal plants would increase sexual reproduction in favorable conditions due to the reduced cost of sexual reproduction. However, Sun et al. [54] reported no discernible pattern of asexual and clonal reproduction along an elevational gradient. In contrast, Liu et al. [31] reported a strong trade-off between sexual and clonal reproduction with intermediate nutrient levels, weaker trade-offs with low nutrient levels, and no trade-offs with high nutrient levels. Matlaga and Horvitz [40] conducted a 2-year study revealing the evidence of trade-offs between reproductive modes but produced no evidence of lower reproductive costs in plants growing in high-light environments. Furthermore, Gaskin et al. [22] found that vegetative versus sexual reproduction in *Convolvulus arvensis* varied significantly across western North America.

Resource allocation to sexual and clonal reproduction in clonal plants can be influenced by the characteristics of the plant itself including size, successional stages and population age [62, 63], as well as environmental conditions such as nutrient levels, light availability, water stress, and elevation gradient [5, 31, 54, 68]. Several mathematical models have predicted that plants allocate more resources to sexual reproduction when resources are scarce or environmental pressure is high, and sexual reproduction should be minimized and clonal reproduction maximized in the most favorable habitats [20, 21]. In some studies such effects have been observed,for instance, van Kleunen [57] reported that *Mimulus guttatus* exhibited significantly greater clonal reproduction in permanently wet conditions than in temporarily wet conditions. Chen et al. [13] reported that sexual reproduction was favored in disturbed habitats with fertile soils, whereas clonal reproduction was favored in stable and competitive habitats. Additionally, [29] reported that plants in deep water allocated more resources to clonal reproduction than plants in shallow or medium-depth water. These reports suggest that most resources are allocated to sexual reproduction in habitats with fluctuating environmental conditions and strong competition, whereas clonal growth is dominant in stable habitats [80].

Climate change results in extreme weather events (e.g. heat waves, floods and drought), which are predicted to increase in frequency, intensity and duration over the years [25]. Global warming and changes in precipitation patterns are two crucial components of climate change. Over the past few decades, anthropogenic activities have resulted in an average global temperature increase of 0.85 °C compared to preindustrial temperatures [25]. Inter- and intra-annual variability in precipitation patterns and the frequency of extreme precipitation events are predicted to increase in many regions [1, 16, 44, 55]. Such warming and altered precipitation events have substantial effects on plant growth and population persistence, especially in inland arid and semi-arid areas, where precipitation is generally sparse and irregular. Although the effects of warming on plants have received attention, [2, 7, 37, 38, 42, 48, 49, 64, 81] and numerous studies have investigated the effects of warming and precipitation on plant traits (often within the context of drought) [15, 23, 24, 50] only a few have investigated the combined effects of simultaneous warming and precipitation on individual plant species [8, 24]. Furthermore, to our knowledge no study has investigated the combined effects of warming and increased precipitation on both sexual and clonal reproduction in clonal plants. In this study, we investigated how warming, increased precipitation, and interactions between these two factors influenced the sexual and clonal reproduction in the clonal plant *Stipa breviflora*.

*S. breviflora* (Poaceae) is a dominant perennial tillerous clonal plant that is widely distributed in desert steppe regions. It has two different types of ramets: reproductive ramets (tillers with an inflorescence or infructescence), which can produce seeds to maintain sexual reproduction, and vegetative ramets (tillers without an inflorescence or infructescence during the whole growth season), which participatess in clonal reproduction. The flowering and fruiting period of *S. breviflora* is from May to July [19], coinciding with its rapid growth period after the onset of spring. A previous study found that the number of foliage branches (vegetative ramets) significantly surpassed those under moderate and heavy grazing conditions, whereas the number of reproductive branches (reproductive ramets) notably decreased under heavy grazing compared to that under no and moderate grazing conditions [32]. Warming and increased precipitation are both expected in desert steppe regions in China, [11, 25], and they may play important roles in the life history of plant species. In the current study sexual and clonal reproduction responses to warming in combination with increased precipitation were investigated in *S. breviflora* via experiments involving increases in precipitation and warming. We specifically address the following questions: (1) Is there a trade-off between sexual and clonal reproduction in *S. breviflora*? (2) How does *S. breviflora* adapt to rapid climate change to ensure the survival of its population by balancing sexual and clonal reproduction? and (3) Do altered precipitation and temperature promote sexual reproduction in *S. breviflora*?

## Results

### Effects of warming and increased precipitation on whole plants

**Fig. 1:**
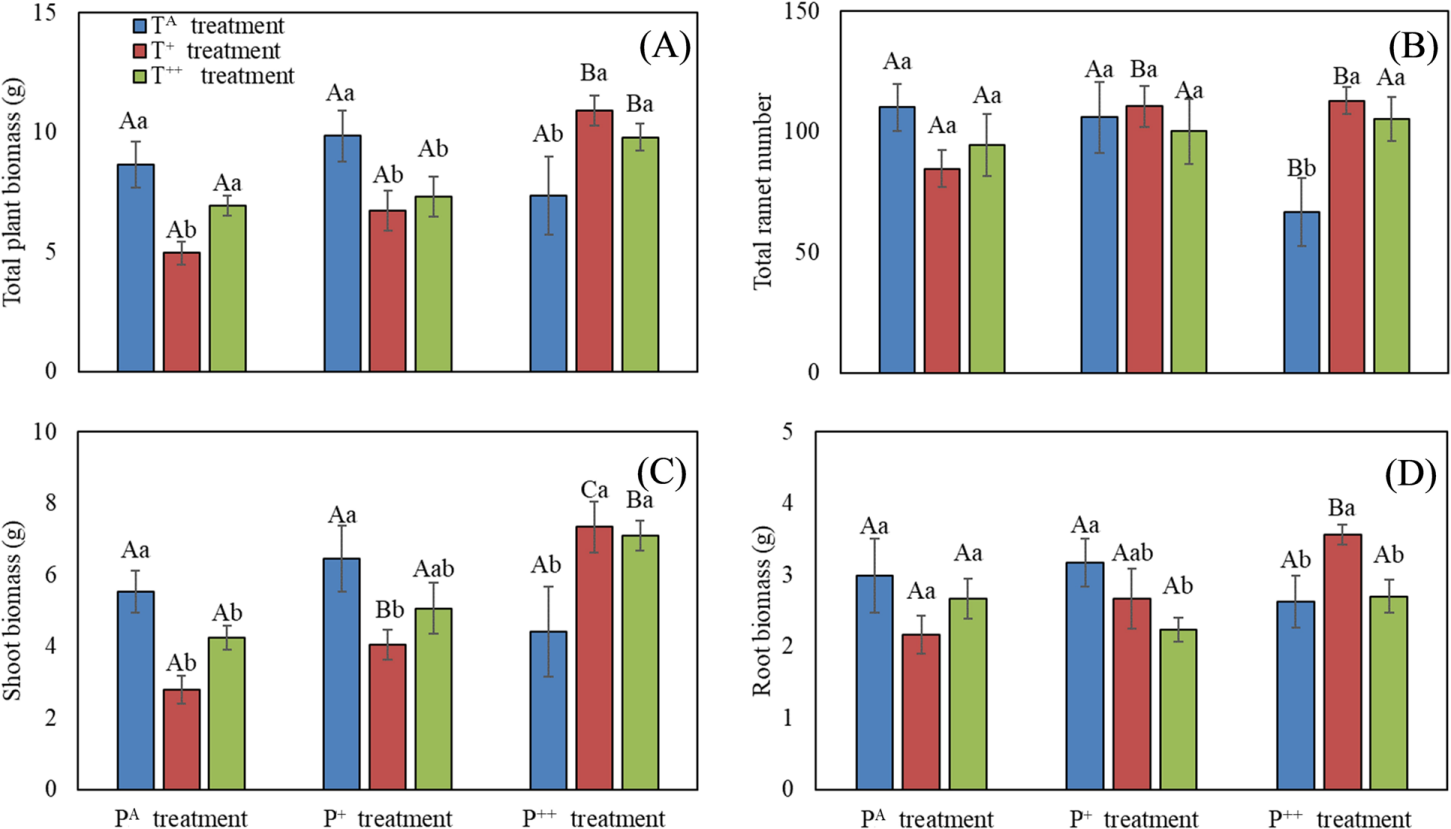
Effects of warming and increased precipitation on total plant biomass (**A**), ramets number (**B**), shoot biomass (**C**), and root biomass (**D**) in *Stipa breviflora*. Different capital letters represent significant differences among three increased precipitation treatments, and different lowercase letters represent significant differences among three warming treatments at *P* < 0.05

Precipitation significantly affected total plant (F_72_ = 5.86, *P* = 0.005) and shoot (F_72_ = 6.71, *P* = 0.003) biomass, but had no effect on total ramet number, root biomass or caryopsis biomass (Table 1). In the T^A^ treatment, precipitation reduced the total number of ramets, but had no effect on total biomass or its allocation. In the T^+^ treatment, precipitation increased both the total number of ramets and biomass, including shoot and root biomass. In the T^++^ treatment, precipitation increased the total and shoot biomass, but had no effects on total ramet number or root biomass (Fig. 1).

**Table 1:** Effects of warming and increased precipitation on plant traits in *Stipa breviflora*

Plant traits	R^2^	Precipitation (P)			Warming (W)			P × W		
		F	*P*	Partial η^2^	F	*P*	Partial η^2^	F	*P*	Partial η^2^
**Ramet number**										
Total ramet number	0.236	0.76	0.473	0.033	0.42	0.659	0.018	2.88	**0.033**	0.204
Reproductive ramet number	0.345	3.76	**0.031**	0.143	3.37	**0.043**	0.130	2.36	0.067	0.173
Vegetative ramet number	0.192	1.03	0.365	0.044	0.95	0.393	0.041	1.68	0.172	0.130
Ratio of reproductive ramet number to total ramet number	0.213	2.06	0.139	0.084	3.41	**0.042**	0.132	0.31	0.872	0.027
**Biomass**										
Total plant biomass	0.439	5.86	**0.005**	0.207	1.09	0.344	0.046	5.33	**0.001**	0.321
Shoot biomass	0.456	6.71	**0.003**	0.230	1.13	0.333	0.048	5.51	**0.001**	0.329
Biomass of reproductive ramets	0.449	7.99	**0.001**	0.262	2.92	0.064	0.115	3.73	**0.011**	0.249
Biomass of vegetative ramets	0.298	0.42	0.662	0.018	0.80	0.455	0.034	4.17	**0.006**	0.270
Ratio of reproductive ramet shoot biomass to total shoot biomass	0.374	4.45	**0.017**	0.165	5.58	**0.007**	0.199	1.71	0.165	0.132
Biomass of single reproductive ramet	0.563	11.27	** < 0.001**	0.334	12.16	** < 0.001**	0.351	2.81	**0.037**	0.200
Biomass of single vegetative ramet	0.138	1.10	0.34	0.047	0.96	0.391	0.041	0.78	0.55	0.065
Root biomass	0.246	0.98	0.382	0.042	1.19	0.312	0.050	2.58	**0.050**	0.187
Caryopsis biomass	0.33	0.63	0.539	0.052	3.84	**0.037**	0.250	0.42	0.664	0.068
**Plant height and leaf traits**										
Reproductive ramet height	0.586	15.74	** < 0.001**	0.412	11.15	** < 0.001**	0.331	2.48	0.057	0.181
Vegetative ramet height	0.229	2.78	0.073	0.110	0.45	0.640	0.020	1.73	0.160	0.133

In contrast, warming significantly affected caryopsis biomass (F_72_ = 3.84, *P* = 0.037), but had no effect on total plant biomass, shoot and root biomass, or ramet number (Table 1). With ambient precipitation, warming reduced the total biomass and shoot biomass. In the P^+^ treatment, warming reduced the total, shoot, and root biomass, but had no effect on total ramet number. In the P^++^ treatment, warming increased the total ramet number and the total biomass and shoot biomass, and it initially increased then decreased root biomass (Fig. 1).

Precipitation had no effect on caryopsis biomass, whereas warming significantly reduced it (F_72_ = 3.84, *P* = 0.037, Table 1, Additional file 1: Fig. 2). There was significant interaction between the effects of precipitation and warming on total ramet number (F_72_ = 2.88, *P* = 0.033), total plant biomass (F_72_ = 5.33, *P* = 0.001), total shoot biomass (F_72_ = 5.51, *P* = 0.001) and root biomass (F_72_ = 2.58, *P* = 0.050, Table 1).

### Effects of warming and increased precipitation on reproductive and vegetative ramets

There were no relationships between the number (F_72_ = 0.095, *P* = 0.429) and shoot biomass (F_72_ = -0.177, *P* = 0.140) of reproductive ramet and those of vegetative ramets.

**Fig. 2:**
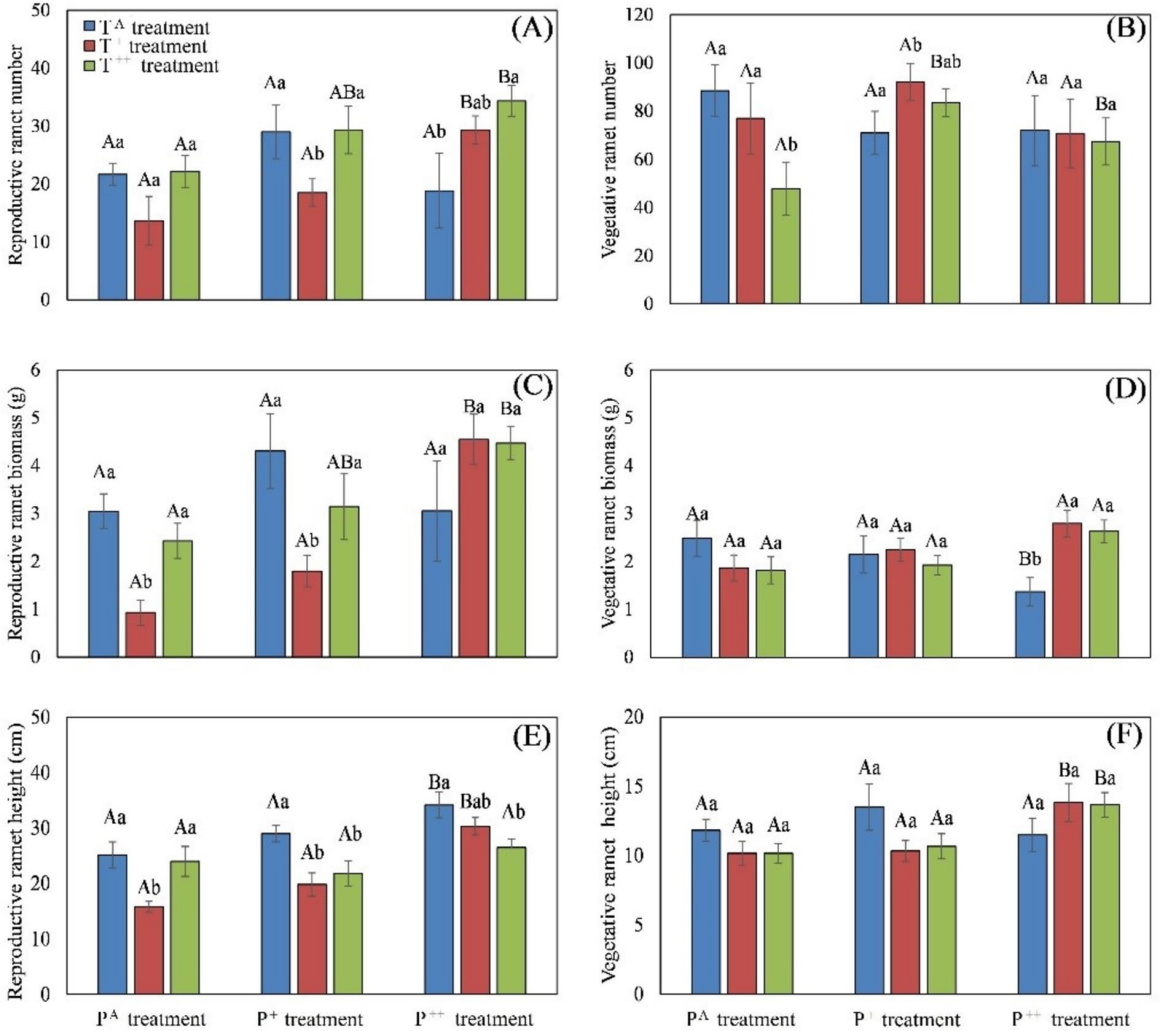
Effects of warming and increased precipitation on reproductive ramet number (**A**), vegetative ramets number (**B**), reproductive ramet shoot biomass (**C**), vegetative ramet shoot biomass (**D**), reproductive ramet height (**E**) and vegetative ramet height (**F**) in *Stipa breviflora*. Different capital letters represent significant differences among three increased precipitation treatments, and different lowercase letters represent significant differences among three warming treatments at *P* < 0.05

Precipitation increased the number, shoot biomass and height of reproductive ramets in the T^+^ treatment. Similarly, in the T^++^ treatment precipitation increased the number and shoot biomass of reproductive ramets. And in the T^A^ treatment, precipitation increased the height of reproductive ramets (Fig. 2). Conversely, T^+^ treatment reduced the number, shoot biomass and height of reproductive ramets in the P^A^ and P^+^ treatments, but had no effect on these parameters in the P^++^ treatment. T^++^ treatment increased the number of reproductive ramets () in the P^++^ treatment, and reduced the height of reproductive ramets in the P^+^ and P^++^ treatments (Fig. 2).

Precipitation reduced both the number and shoot biomass of vegetative ramets in the T^A^ treatment, increased the height and shoot biomass of vegetative ramets in the T^+^ treatment, and increased the number and height of vegetative ramets in the T^++^ treatment (Fig. 2). Both T^+^ and T^++^ treatments increased the shoot biomass of vegetative ramets in the P^++^ treatment, and T^++^ treatment also reduced the number of vegetative ramets in the P^A^ treatment (Fig. 2).

**Fig. 3:**
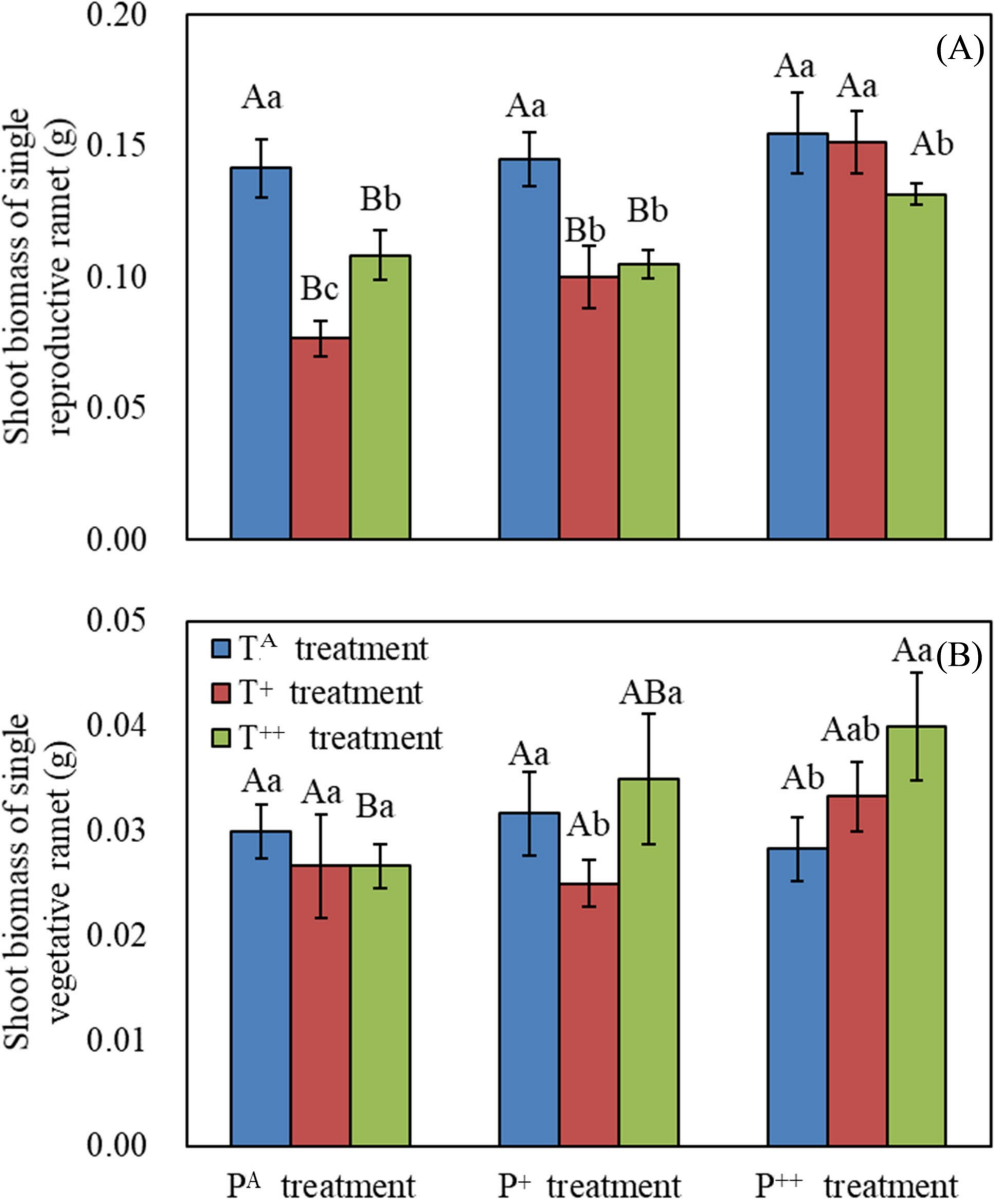
Effects of warming and increased precipitation on mean shoot biomass of single reproductive ramet (**A**) and of single vegetative ramet (**B**) in *Stipa breviflora*. Different capital letters represent significant differences among three increased precipitation treatments, and different lowercase letters represent significant differences among three warming treatments at *P* < 0.05

Both increased precipitation and warming had significant effects on the number (F_72_ = 3.76, *P* = 0.031; F_72_ = 3.37, *P* = 0.043; respectively) and height (F_72_ = 15.74, *P* < 0.001; F_72_ = 11.15, *P* < 0.001; respectively) of reproductive ramets, as well as the shoot biomass of single reproductive ramets (F_72_ = 11.27, *P* < 0.001; F_72_ = 12.16, *P* < 0.001; respectively; Table 1). Increased precipitation significantly affected the shoot biomass of reproductive ramets (F_72_ = 7.99, *P* = 0.001), while warming did not (Table 1). There were significant interactions between the effects of both precipitation and warming on the shoot biomass of reproductive ramets, as well as the shoot biomass of single reproductive ramets (Table 1). Warming reduced the shoot biomass of single reproductive ramets, whereas precipitation increased this in both the T^+^ and T^++^ conditions (Fig. 3).

Neither precipitation nor warming had any significant effects on the total number, height, or shoot biomass, nor on the shoot biomass of individual vegetative ramets (Table 1). However, precipitation did increase the shoot biomass of single vegetative ramets in the T^+^ and T^++^ treatments. Additionally, T^+^ treatment reduced the shoot biomass of single vegetative ramets in the P^+^ treatment, while T^++^ treatment increased this in the P^++^ treatment (Fig. 3).

**Fig. 4:**
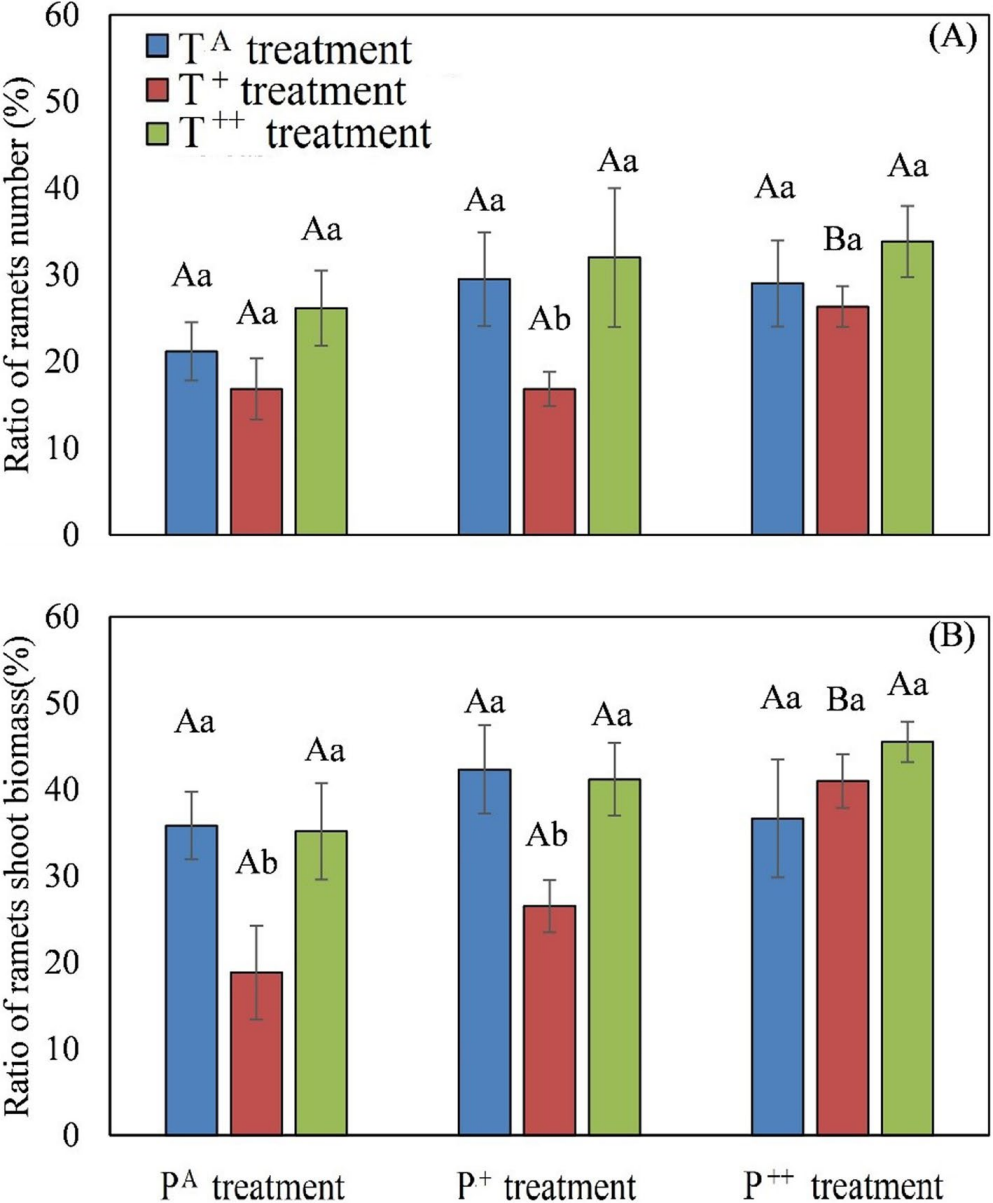
Effects of warming and increased precipitation on the ratio of number (**A**) and the ratio of shoot biomass (**B**) of reproductive ramets to total ramet number and total shoot biomass in *Stipa breviflora*. Different capital letters represent significant differences among three increased precipitation treatments, and different lowercase letters represent significant differences among three warming treatments at *P* < 0.05

Precipitation significantly affected the ratio of reproductive ramets shoot biomass to total shoot biomass (F_72_ = 4.45, *P* = 0.017), and warming significantly affected both the ratio of reproductive ramet number to total ramet number (F_72_ = 3.41, *P* = 0.042) and the ratio of reproductive ramet shoot biomass to total shoot biomass (F_72_ = 5.58, *P* = 0.007; Table 1). P^++^ treatment increased both the ratio of reproductive ramet number to total ramet number and the ratio of reproductive ramet shoot biomass to total shoot biomass under the T^+^ condition, and there were insignificant increases under the T^A^ and T^++^ conditions (Fig. 4). Compared to the control treatment, T^+^ treatment decreased the ratio of reproductive ramet number to total ramet number under the P^+^ condition, as well as the ratio of the reproductive ramet shoot biomass to total shoot biomass under the P^A^ condition and the P^+^ condition (Fig. 4). T^++^ treatment had no effects on either the ratio of reproductive ramet number to total ramet number or the ratio of reproductive ramet shoot biomass to total shoot biomass ramets under any of the three tested precipitation conditions (Fig. 4).

## Discussion

This study presents experimental evidence on how the combination of warming and precipitation affects sexual and clonal reproduction in *S. breviflora*, a dominant species in desert steppe regions. A trade-off between sexual and clonal reproduction is generally assumed in clonal plants because resources availability is frequently limited. However, previous studies have yielded mixed results; some have detected negative correlations between sexual and clonal reproduction [29, 40, 54], while others have not [6, 43, 58]. Our results indicate that both warming and increased precipitation significantly increased reproductive ramet number and shoot biomass, with no effect on vegetative ramet number or shoot biomass. There was no relationship between the number or shoot biomass of reproductive and vegetative ramets. This suggests that there is no trade-off between sexual and clonal reproduction in the clonal grass *S. breviflora*, despite the effects of warming and increased precipitation on plant growth.

Warming generally promotes plant growth and biomass accumulation by extending the growing season, enhancing soil nutrient availability and plant photosynthesis rates, and modifying water-use strategies [14, 26, 30, 71]. However, these effects of warming on plant growth may depend on precipitation because warming can increase water stress by reducing soil moisture and accelerating evapotranspiration [30, 45, 77, 78]. In situations where water is limited, the negative effects of water stress may outweigh the beneficial effects of warming. In the current study, warming reduced total, shoot, and root biomass in *S. breviflora* with ambient and moderately increased precipitation, but warming increased the parameters with highly increased precipitation (Fig. 1). These results suggest that warming may have a negative net impact on plant growth, resulting in reduced plant biomass accumulation, especially in regions with scarce precipitation and/or in years with below-average precipitation, which is consistent with previous studies [3, 30, 46]. The negative effects of warming on functional traits (morphological and physical) may particularly affect *S. breviflora*, as it is a C_3_ plant [33]. Warming reduced the importance value of *S. breviflora*, while increased those of annual herbs in the temperate desert steppe, suggesting *S. breviflora* will lose its status as the constructive species in the context of future climate change [36]. Conversely, increased precipitation may mitigate the negative effects of warming on plant growth and instead have positive effects on earlier green-up and later senescence [37]. Similar results have been observed in *S. grandis*, whose biomass was enhanced by moderate warming and additional precipitation, but declined drastically with high temperature and drought [53].

Warming had significant effects on the sexual reproduction of *S. breviflora*, affecting the number of reproductive ramets, the ratio of reproductive ramet number to the total ramet number, and the reproductive ramet shoot biomass (Table 1). In the present study, 1.5 °C of warming significantly reduced the growth of reproductive ramets with ambient and moderately increased precipitation (lower ramet number, height, and shoot biomass), but increased reproductive ramet number and shoot biomass with highly increased precipitation (Fig. 2). Furthermore, warming significantly reduced the size of reproductive ramets with ambient and moderately increased precipitation, but had no effect with highly increased precipitation (Fig. 3). These results suggest that warming might inhibit or promote reproductive ramet growth depending on precipitation conditions [34], leading to shifts in investment between sexual and clonal reproduction in *S. breviflora*. The water stress induced by warming forced clonal plants to reduce investment in sexual reproduction; however, increasing precipitation could compensate for the adverse effects of warming on sexual reproduction in *S. breviflora* [51].

Precipitation plays a crucial role in plant growth by influencing soil water content, especially in arid and semi-arid regions characterized by sparse and irregular precipitation. Increased precipitation is known to enhance plant growth and photosynthesis, while water deficit has the opposite effects [53, 66]. Our study is partially in line with these general assumptions. Precipitation significantly increased both total plant biomass and shoot biomass with temperature increases of 1.5 °C and 3.0 °C, and significantly increased root biomass with 1.5 °C of warming. However, it had no effects on plant performance in ambient temperature (Fig. 1). This suggests that excess water cannot be fully utilized by plants in ambient temperature conditions in this semiarid region [74, 75]. Increased precipitation, in conjunction with warming, promoted plant growth in *S. breviflora*, it exhibited significant association with total plant biomass, shoot biomass and root biomass (Table 1).

Precipitation has contrasting effects on different ramet types in *S. breviflora*; in ambient temperature conditions, it significantly reduced ramet number and shoot biomass of vegetative ramets. However, in conjunction with temperature increases of 1.5 °C and 3.0 °C, precipitation increased shoot biomass and the number and height of reproductive ramets. Precipitation also increased both the ratio of reproductive ramet number to total ramet number and the ratio of reproductive ramet shoot biomass to total shoot biomass in conjunction with 1.5 °C warming. Thus, the combination of precipitation and warming is more beneficial to sexual reproduction than clonal reproduction in *S. breviflora*, in accordance with the prediction that clonal plants increase sexual reproduction under favorable conditions due to the reduced cost of sexual reproduction [35].

Clonal reproduction may be more advantageous than sexual reproduction in resource-limited and highly disturbed environments [17, 69]. Warming and increased precipitation significantly affected the number, biomass and height of reproductive ramets, as well as the shoot biomass of single reproductive ramets. In contrast, vegetative ramets were less affected by warming and precipitation changes. This suggests that reproductive ramets are more sensitive to climate change than vegetative ramets in the clonal plant *S. breviflora*. The significant reduction in caryopsis biomass with both 1.5 °C and 3.0 °C of warming (Additional file 1: Fig. 2) further indicates the significant increase in the cost of sexual reproduction conferred by warming. These findings highlight that clonal reproduction may serve as an effective strategy for clonal plants to cope with climate change ensuring population survival [4].

Notably, the current study was a short-term experiment conducted on a single species. Thus, long-term, multi-species experiments are needed to further investigate how sexual and clonal reproduction change in response to climate change. Moreover, it is important to consider the potential limitations associated with the use of open-top chambers (OTCs) for applying treatment. OTCs may introduce unwanted ecological effects, such as chamber overheating and altered light, soil moisture, and wind speed, as well as increased air and soil temperature [39], however, increased overheating and evaporation are considered inseparable consequences in a warming climate [47]. Climate change may also lead to changes in addition to warming and alterations in rainfall, including extreme events, such as earlier or later frosts, extremely high or low temperatures, and longer drought periods. Therefore, more studies are necessary to investigate the potential impacts of climate change, especially extreme climate events, on plant performance.

## Conclusions

This study provides experimental evidence that there is no trade-off between sexual and clonal reproduction in *S. breviflora*. The findings demonstrate that both sexual and clonal reproductions in this species are influenced by the combined effects of warming and increased precipitation, stimulating realistic conditions in a desert steppe ecosystem in northern China. Increased precipitation promotes sexual reproduction, whereas the effects of warming on reproductive and/or vegetative ramets in *S. breviflora* may vary depending on precipitation conditions. Reproductive ramets of *S. breviflora* were more sensitive to simulated climate change than vegetative ramets in this study. This suggests that clonal plants, with the capacity for both sexual and clonal reproduction, can effectively adapt to climate change through clonal reproduction, ensuring the renewal and survival of the population. To gain a more comprehensive understanding of the effects of climate change on sexual and clonal reproduction in clonal plants, further long-term, multi-species experiments are needed.

## Methods

### Study site

The study was conducted at the Siziwang Banner Research Station, of the Inner Mongolia Academy of Agriculture and Animal Husbandry Sciences (Siziwang Station). The Siziwang Station (41°47.28’ N, 111°53.77’E, 1450 m a.s.l.) is located in western Inner Mongolia, China, and has a temperate continental climate characterized by a short growing season and a long cold winter. The mean annual temperature was 3.6 °C, with a minimum mean monthly temperature of -15.1 °C (January) and a maximum mean monthly temperature of 19.6 °C (July). Mean annual precipitation was 280 mm, and most precipitation occurred between June and September (data from a meteorological station at Siziwang Station, 1985–2014).

Desert grassland is the dominant ecosystem in the study area, and it is dominated by *S. breviflora* Griseb., *Artemisia frigida* Willd., and *Cleistogenes songorica* (Roshev.) Ohwi, accompanied by *Convolvulus ammannii* Desr., *Heteropappus altaicus* Willd., *Caragana stenophylla* Pojark., *Caragana intermedia* Kuang et H. C. Fu and *Leymus chinensis* (Trin.) Tzvel..

### Plant material

*Stipa breviflora* Griseb. (Poaceae) is a perennial bunchgrass that grows to approximately 20–60-cm in height. It is distributed in gravel and rocky slopes in various provinces (Gansu, Hebei, Ningxia, Qinghai, Shaanxi, Shanxi and Sichuan), and the autonomous regions (Inner Mongolia, Xinjiang and Xizang) of China, as well as in Kazakhstan, Kyrgyzstan, Mongolia, Nepal, Tajikistan and Uzbekistan. *S. breviflora* is commonly found in desert steppe regions and serves as a spring forage grass [19].

*S. breviflora* is a fibrous-rooted plant, with a root system typically reaching a length of approximately 15 cm. As a tillerous clonal plant, *S. breviflora* can reproduce new ramet through tillering, which is its most important mode of reproduction [12, 79]. This includes both reproductive ramets (tillers with an inflorescence, 30–40 cm high) and vegetative ramets (tillers without an inflorescence, 10–15 cm high). The growth of all ramets begins in mid-April, with differentiation in late May. Vegetative ramets grow from mid-April to mid-September, peaking from late May to mid- June. Reproductive ramets enter the reproductive stage in late May and the post-fruiting nutritional stage in late June [79]. Vegetative ramets die in each period of the growing season, especially in the reproductive period (May to June). One of the main reasons for this is that reproductive ramets consume a large amount of nutrients and have limited photosynthetic capacity (3–4 leaves per ramet). They rely on the photosynthetic products of closely associated vegetative ramets, leading to the death of the latter [79].

### Experimental design

In early May 2017, an area measuring a 50 × 50 m located approximately 200 m east of Siziwang Station, where *S. breviflora* was growing was chosen as the source of experimental materials. There were approximately 17 *S. breviflora* clumps per square meter. A total of 72 similar-sized clumps of *S. breviflora* were collected. The distance between each clump was more than 2 m. These clumps consisted of 34–46 tillers, which were newly budded after winter withering and had the potential to differentiate into vegetative ramets or reproductive ramets in late May. At the time of collection, it was very difficult to identify whether ramets were reproductive or vegetative. Therefore, randomly selected tillers were cut off with scissors in the standardization process to ensure that each clump was the same size (approximately 4 cm in diameter). After standardization, each clump consisted of thirty 10-cm-high tillers and thirty 10-cm-long fibrous roots. A total of 72 clumps were then planted into individual plastic containers measuring 18 cm in height and 25 cm in diameter. The soil used in the experiment was collected from the habitat, and roots and stones were removed using a 2-mm sieve. The soil had an average total P content of 0.403 ± 0.007 g/kg (mean ± S.E.), total N content of 1.738 ± 0.356 g/kg, total C content of 15.839 ± 0.345 g/kg, and a soil pH of 8.065 ± 0.245. After 2 weeks of recovery, on May 28, 2017, all containers were placed on the Warming and Precipitation Enhancement Platform in Siziwang Station (Additional file 1: Fig 3) and randomly assigned to one of nine treatments in a factorial design, including three levels of precipitation and three levels of warming. The precipitation treatments represented strongly increased precipitation (P^++^), moderately increased precipitation (P^+^), and ambient precipitation (P^A^), respectively. The warming treatments represented an increase of approximately 3 °C (T^++^), an increase of approximately 1.5 °C (T^+^), and ambient temperature (T^A^), respectively. There were eight replicates of each treatment. These treatments represent realistic scenarios for the region with slight overall increases in precipitation [11] and warming [25].

Precipitation conditions were produced through artificial watering. For the P^A^ treatment, 350.0 mL water was administered every 3 days, simulating ambient precipitation in the growing season (a mean of 174 mm precipitation from June to August in the years 1985–2014, according to meteorological data generated by the Siziwang Station). For the P^+^ treatment, 437.5 mL water was administered every 3 days, increasing precipitation by 25%. For the P^++^ treatment, 525.0 mL water was administered every 3 days, increasing precipitation by 50%. All plants were protected from natural rain by a plastic rain film placed 100 cm above them.

Warming conditions were implemented using different open-top chambers (OTCs). For the T^A^ treatment, potted plants were placed outside the OTC, for the T^+^ treatment, potted plants were placed in OTC1, and for the T^++^ treatment, potted plants were placed in OTC2. All OTCs were constructed with a stainless-steel frame and a 5-mm-thick box of tempered glass. OTC1 was 100-cm high, with a regular hexagonal shape 150 cm in length. OTC2 was 230-cm high, with a regular hexagonal shape 150 cm in length. In total, 12 OTC1s and 12 OTC2s were used in the study.

The experimental period lasted from May 28, 2017, to August 28, 2017, totaling92 days. During this period, a total of 88.9 mm of precipitation was recorded, 27.18 mm in June, 30.5 mm in July, and 31.3 mm in August (data from the meteorological station at Siziwang Station). The average ambient air temperature was 22.3 °C, with an average of 54.7% relative air humidity (data were collected by Em50 series data loggers, Decagon Devices, Inc., USA; Additional file 1: Fig 1). In the T^+^ treatment, the average air temperature increased by 1.5 °C to 23.8 °C, and the relative air humidity increased by 1.1% to 55.9% (Additional file 1: Fig 1). In the T^++^ treatment, the average air temperature increased by 3.3 °C to 25.6 °C, and the relative air humidity decreased by 4.2% to 50.5% (Additional file 1: Fig 1). And during the experiment period, the containers were moved weekly to avoid systematic errors caused by location.

### Measurements and analysis

All plants were harvested on August 28, 2017. The tillers in each clump were categorized as either reproductive or vegetative ramets, and the plant clumps were further divided into shoot, root, and caryopsis. Measurements and recordings were made for the number, height, and shoot biomass of reproductive and vegetative ramets in each plant clump. Due to the difficulty of dividing the root biomass into different tillers, this was measured for each clump as a whole. Caryopsis (seeds with palea but without awn) were manually collected from each clump to measure caryopsis biomass. Total ramet number, total plant biomass, total shoot biomass, and the ratios of the number and shoot biomass of reproductive ramets to total ramets number were calculated. Additionally, the shoot biomass of single reproductive/vegetative ramets was calculated by dividing their respective shoot biomass by the number of ramets. All plant structures were weighted after drying at 80 °C for 48 h.

Two-way analysis of variance was used to examine the effects of increased precipitation and warming, as well as possible interactions between the two on ramet number, shoot biomass, height of both reproductive and vegetative ramets, ratios of ramet number and shoot biomass of reproductive ramets to the total ramet number, biomass, and its allocation within the plant clump. We used the partial η^2^ (eta-squared) value to quantify the effect size of different treatments on plant traits, which was defined as SS_A_/(SS_A_ + SS_E_). Tukey’s honestly significant difference test was used for multiple comparisons of the main effects of increased precipitation and warming. Data were log_10_(x + 1) transformed if necessary to satisfy normality and homogeneity of variance. Additionally a two-tailed Pearson’s correlation test was used to analyze the relationships between the number and shoot biomass of reproductive ramets and that of vegetative ramets. All statistical analyses were performed with SPSS version 18.0 (SPSS Inc., United States, 2009).

### Supplementary Information

**Additional file 1: Figure 1.** Air temperature (A) and relative moisture (B) under different warming treatments during the experimental period (from May 28 to August 27, 2017). **Figure 2.** Effects of warming and increased precipitation on caryopsis biomass (mean ± SE) in *Stipa breviflora*. Different capital letters represent significant differences among three increased precipitation treatments, and different lowercase letters represent significant differences among three warming treatments at *P*< 0.05. **Figure 3.** The Warming and Precipitation Enhancement Platform in Siziwang Station.

## Data Availability

All data generated or analysed during this study are included in this published article [and its supplementary information files].

## References

[1] Allan RP, Soden BJ (2008). Atmospheric warming and the amplification of precipitation extremes. Science.

[2] Alzate-Marin AL, Rivas PMS, Galaschi-Teixeira JS, Bonifacio-Anacleto F, Silva CC, Schuster I, et al. Warming and elevated CO2 induces changes in the reproductive dynamics of a tropical plant species. Sci Total Environ. 2021; 768: 144899. 10.1016/j.scitotenv.2020.144899. 10.1016/j.scitotenv.2020.144899 33736351

[3] Bai WM, Wan SQ, Niu SL, Liu WX, Chen QS, Wang QB (2010). Increased temperature and precipitation interact to affect root production, mortality, and turnover in a temperate steppe: implications for ecosystem C cycling. Global Change Biol.

[4] Bam S, Ott JP, Butler JL, Xu L (2022). Belowground mechanism reveals climate change impacts on invasive clonal plant establishment. Sci Rep.

[5] Bengtsson BO, Ceplitis A (2000). The balance between sexual and asexual reproduction in plants living in variable environments. J Evolution Biol.

[6] Bilgin A, Kilinc M, Kutbay HG, Yalcin E, Huseyinova R. Effects of sexual reproduction on growth and vegetative propagation in *Arum maculatum* L. (Araceae): in-situ removal experiments. Pol J Ecol. 2009; 57: 261–288.

[7] Bjorkman AD, Myers-Smith IH, Elmendorf SC, Normand S, Ruger N, Beck PSA (2018). Plant functional trait change across a warming tundra biome. Nature.

[8] Bloor JMG, Pichon P, Falcimagne R, Leadley P, Soussana J (2010). Effects of warming, summer drought, and CO_2_ enrichment on aboveground biomass production, flowering phenology, and community structure in an upland grassland ecosystem. Ecosystems.

[9] Cao XX, Xue W, Lei NF, Yu FH (2022). Effects of clonal integration on foraging behavior of three clonal plants in heterogeneous soil environments. Forests.

[10] Chen D, Ali A, Yong XH, Lin CG, Niu XH, Cai AM (2019). A multi-species comparison of selective placement patterns of ramets in invasive alien and native clonal plants to light, soil nutrient and water heterogeneity. Sci Total Environ.

[11] Chen HP, Sun JQ, Chen XL, Zhou W (2012). CGCM projections of heavy rainfall events in China. Int J Climato.

[12] Cheng SH, Zhang H (1991). A study on ecological and biological characteristics of *Stipa brevifolia*. Grassl Pratacul.

[13] Chen XS, Li YF, Xie YH, Deng ZM, Li X, Li F (2015). Trade-off between allocation to reproductive ramets and rhizome buds in *Carex brevicuspis* populations along a small-scale elevational gradient. Sci Rep-UK.

[14] Danby RK, Hik DS (2007). Responses of white spruce (*Picea glauca*) to experimental warming at a subarctic alpine treeline. Global Change Biol.

[15] Drebenstedt I, Schmid I, Poll C, Marhan S, Kahle R, Kandeler E (2020). Effects of soil warming and altered precipitation patterns on photosynthesis, biomass production and yield of barley. J Appl Bot Food Qual.

[16] Durack PJ, Wijffels SE, Matear RJ (2012). Ocean salinities reveal strong global water cycle intensification during 1950 to 2000. Science.

[17] Endo H, Sugie T, Yonemori Y, Nishikido Y, Moriyama H, Ito R (2021). Vegetative reproduction is more advantageous than sexual reproduction in a canopy-forming clonal macroalga under ocean warming accompanied by oligotrophication and intensive herbivory. Plants-Basel.

[18] Eriksson O, de Kroon H, van Groenendael J (1997). Clonal life histories and the evolution of seed recruitment. The ecology and evolution of clonal plants.

[19] Flora of China Editorial Committee (2006). Flora of China,.

[20] Fu LK, Wang SC, Liu ZG, Nijs I, Ma KP, Li ZQ (2010). Effects of resource availability on the trade-off between seed and vegetative reproduction. J Plant Ecol.

[21] Gardner SN, Mangel M (1999). Modeling investments in seeds, clonal offspring, and translocation in a clonal plant. Ecology.

[22] Gaskin JF, Cortat G, West NM (2023). Vegetative versus sexual reproduction varies widely in *Convolvulus arvensis* across western North America. Biol Invasions.

[23] Gilgen AK, Buchmann N (2009). Response of temperate grasslands at different altitudes to simulated summer drought differed but scaled with annual precipitation. Biogeosciences.

[24] Hamann E, Kesselring H, Stocklin J (2018). Plant responses to simulated warming and drought: a comparative study of functional plasticity between congeneric mid and high elevation species. J Plant Ecol.

[25] IPCC. Special report on global warming of 1.5℃. UK: Cambridge University Press; 2018.

[26] Klady RA, Henry GHR, Lemay V (2011). Changes in high arctic tundra plant reproduction in response to long-term experimental warming. Global Change Biol.

[27] Klimeš L, Klimešová J, Hendricks R, van Groenendael J. Clonal plant architecture: a comparative analysis of form and function. In: de Kroon H, van Groenendael J, editors. The ecology and evolution of clonal plants. Leiden: Backbuys Publishers; 1997. p. 1–29.

[28] Kocot D, Sitek E, Nowak B, Kolton A, Stachurska-Swakon A, Towpasz K (2022). The effectiveness of the sexual reproduction in selected clonal and nonclonal species of the genus ranunculus. Biology-Basel.

[29] Li L, Lan ZC, Chen JK, Song ZP. Allocation to clonal and sexual reproduction and its plasticity in *Vallisneria spinulosa* along a water-depth gradient. Ecosphere. 2018; 9: e02070. 10.1002/ecs2.2070.

[30] Lin DL, Xia JY, Wan SQ (2010). Climate warming and biomass accumulation of terrestrial plants: a meta-analysis. New Phytol.

[31] Liu F, Chen JM, Wang QF (2009). Trade-offs between sexual and asexual reproduction in a monoecious species *Sagittaria pygmaea* (Alismataceae): the effect of different nutrient levels. Plant Syst Evol.

[32] Liu W (2020). Individual reproductive strategy of *Stipa breviflora* under different grazing pressures. Grassl Sci.

[33] Liu XZ, Li Y, Zhang Y, Su Q, Feng T, Song Y (2022). N-15 natural abundance of C3 and C4 herbaceous plants and its response to climatic factors along an Agro-Pastoral Zone of Northern China. Plants-Basel.

[34] Liu Y, Bachofen C, Lou YJ, Ding Z, Jiang M, Lu XG (2021). The effect of temperature changes and K supply on the reproduction and growth of *Bolboschoenus planiculmis*. J Plant Ecol.

[35] Loehle C (1987). Partitioning of reproductive effort in clonal plants - a benefit-cost model. Oikos.

[36] Lv GY, Wang ZY, Guo N, Xu XB, Liu PB, Wang CJ. Status of *Stipa breviflora* as the constructive species will be lost under climate change in the temperate desert steppe in the future. Ecol Indicat. 2021; 126: 107715. 10.1016/j.ecolind.2021.107715.

[37] Ma PF, Zhao JX, Zhang HZ, Zhang L, Luo TX. Increased precipitation leads to earlier green-up and later senescence in Tibetan alpine grassland regardless of warming. Sci Total Environ. 2023;871. 10.1016/j.scitotenv.2023.162000. 10.1016/j.scitotenv.2023.162000 36739031

[38] Maluf RP, Alzate-Marin AL, Silva CC, Pansarin LM, Bonifacio-Anacleto F, Schuster I, et al. Warming and soil water availability affect plant-flower visitor interactions for *Stylosanthes capitate*, a tropical forage legume. Sci Total Environ. 2022; 817: 152982. 10.1016/j.scitotenv.2022.152982. 10.1016/j.scitotenv.2022.152982 35031369

[39] Marion GM, Henry GHR, Freckman DW, Johnstone J, Jones G, Jones MH (1997). Open-top designs for manipulating field temperature in high-latitude ecosystems. Global Change Biol.

[40] Matlaga DP, Horvitz CC (2015). Large size and high light do not lower the cost of reproduction for the neotropical herb *Goeppertia Marantifolia*. Am J Bot.

[41] Mattingly KZ, Day CTC, Rauschert ESJ, Tayal A, Hovick SM (2023). Genetic and morphological comparisons of lesser celandine (*Ficaria verna*) invasions suggest regionally widespread sexual reproduction. Biol Invasion.

[42] Memmott J, Craze PG, Waser NM, Price MV (2007). Global warming and the disruption of plant-pollinator interactions. Ecol Lett.

[43] Mendez M (1999). Effects of sexual reproduction on growth and vegetative propagation in the perennial geophyte *Arum italicum* (Araceae). Plant Biol.

[44] Min SK, Zhang XB, Zwiers FW, Hegerl GC (2011). Human contribution to more intense precipitation extremes. Nature.

[45] Niu SL, Li ZX, Xia JY, Han Y, Wu MY, Wan SQ (2008). Climatic warming changes plant photosynthesis and its temperature dependence in a temperate steppe of northern China. Environ Exp Bot.

[46] Peñuelas J, Gordon C, Llorens L, Nielsen T, Tietema A, Beier C (2004). Nonintrusive field experiments show different plant responses to warming and drought among sites, seasons, and species in a north–south European gradient. Ecosystems.

[47] Peñuelas J, Sardans J, Estiarte M, Ogaya R, Carnicer J, Coll M (2013). Evidence of current impact of climate change on life: a walk from genes to the biosphere. Global Change Biol.

[48] Prevey JS, Rixen C, Ruger N, Hoye TT, Bjorkman AD, Myers-Smith IH (2019). Warming shortens flowering seasons of tundra plant communities. Nat Ecol Evol.

[49] Root TL, Price JT, Hall KR, Schneider SH, Rosenzweig C, Pounds JA (2003). Fingerprints of global warming on wild animals and plants. Nature.

[50] Rosenblatt AE (2018). Shifts in plant nutrient content in combined warming and drought scenarios may alter reproductive fitness across trophic levels. Oikos.

[51] Shi LN, Lin ZR, Wei XT, Peng CJ, Yao ZY, Han B (2022). Precipitation increase counteracts warming effects on plant and soil C:N: P stoichiometry in an alpine meadow. Front Plant Sci.

[52] Song MH, Dong M (2002). Clonal plants and plant species diversity in wetland ecosystems in China. J Veg Sci.

[53] Song XL, Wang YH, Lv XM (2016). Responses of plant biomass, photosynthesis and lipid peroxidation to warming and precipitation change in two dominant species (*Stipa grandis* and *Leymus chinensis*) from North China Grasslands. Ecol Evol.

[54] Sun SC, Gao XM, Cai YL (2001). Variations in sexual and asexual reproduction of *Scirpus mariqueter* along an elevational gradient. Ecol Res.

[55] Tu JQ, Lu E (2022). Understanding the uncertainty of the long-term precipitation trend under global warming through the water cycle. Int J Climatol.

[56] van Drunen WE, Dorken ME (2012). Trade-offs between clonal and sexual reproduction in *Sagittaria latifolia* (Alismataceae) scale up to affect the fitness of entire clones. New Phytol.

[57] van Kleunen M (2007). Adaptive genetic differentiation in life-history traits between populations of *Mimulus guttatus* with annual and perennial life-cycles. Evol Ecol.

[58] Verburg RW, During HJ (1998). Vegetative propagation and sexual reproduction in the woodland understorey pseudo-annual *Circaea lutetiana* L. Plant Ecol.

[59] Wan JZ, Wang CJ, Yu FH (2019). Large-scale environmental niche variation between clonal and non-clonal plant species: roles of clonal growth organs and ecoregions. Sci Total Environ.

[60] Wang JY, Xu TT, Wang Y, Li GY, Abdullah I, Zhong ZW, et al. A meta-analysis of effects of physiological integration in clonal plants under homogeneous vs. heterogeneous environments. Func Ecol. 2021; 35: 578–589. doi: 10.1111/1365-2435.13732.

[61] Wang MZ, Li HL, Liu CX, Dong BC, Yu FH (2022). Adaptive plasticity in response to light and nutrient availability in the clonal plant Duchesnea indica. J Plant Ecol.

[62] Weppler T, Stöcklin J (2005). Variation of sexual and clonal reproduction in the alpine *Geum reptans* in contrasting altitudes and successional stages. Basic Appl Ecol.

[63] Weppler T, Stoll P, Stöcklin J (2006). The relative importance of sexual and clonal reproduction for population growth in the long-lived alpine plant *Geum reptans*. J Ecol.

[64] Wolkovich EM, Cook BI, Allen JM, Crimmins TM, Betancourt JL, Travers SE (2012). Warming experiments under-predict plant phenological responses to climate change. Nature.

[65] Wu GL, Hu TM, Liu ZH. Trade-off of sexual and asexual recruitment in a dominant weed Ligularia virgaurea (Maxim.) in alpine grasslands (China). Pol J Ecol. 2010;58:81–6.

[66] Wu Z, Dijkstra P, Koch GW, PeÑuelas J, Hungate BA (2011). Responses of terrestrial ecosystems to temperature and precipitation change: a meta-analysis of experimental manipulation. Global Change Biol.

[67] Xiao YA, Dong M, Wang N, Lan LL. Effects of organ removal on trade-offs between sexual and clonal reproduction in the stoloniferous herb Duchesnea indica. Plant Species Biol. 2016;50–4. 10.1111/1442-1984.12077.

[68] Xiao Y, Tang JB, Qing H, Zhou CF, Kong WJ, An SQ (2011). Trade-offs among growth, clonal, and sexual reproduction in an invasive plant *Spartina alterniflora* responding to inundation and clonal integration. Hydrobiologia.

[69] Xiao Y, Zhao H, Yang W, Qing H, Zhu CF, Tang JB, et al. Variations in growth, clonal and sexual reproduction of Spartina alterniflora responding to changes in clonal integration and sand burial. Clean-Soil Air Water. 2015;43: 1100−6. 10.1002/clen.201300868.

[70] Xing YP, Wei GW, Luo FL, Li CY, Dong BC, Ji JS (2019). Effects of salinity and clonal integration on the amphibious plant *Paspalum paspaloides*: growth, photosynthesis and tissue ion regulation. J Plant Ecol.

[71] Xu X, Shi Z, Li DJ, Zhou XH, Sherry RA, Luo YQ (2015). Plant community structure regulates responses of prairie soil respiration to decadal experimental warming. Global Change Biol.

[72] Yan X, Wang HW, Wang QF, Rudstam LG (2013). Risk spreading, habitat selection and division of biomass in a submerged clonal plant: Responses to heterogeneous copper pollution. Environ Pollut.

[73] Ye XH, Gao SQ, Liu ZL, Zhang YL, Huang ZY, Dong M (2015). Multiple adaptations to light and nutrient heterogeneity in clonal plant *Leymus secalinus* with a combined growth form. Flora.

[74] Ye XH, Liu ZL, Gao SQ, Cui QG, Liu GF, Du J (2017). Differential plant species responses to interactions of sand burial, precipitation enhancement and climatic variation promote coexistence in Chinese steppe vegetation. J Veg Sci.

[75] Ye XH, Liu ZL, Zhang SD, Gao SQ, Liu GF, Cui QG (2019). Experimental sand burial and precipitation enhancement alter plant and soil carbon allocation in a semi-arid steppe in north China. Sci Total Environ.

[76] Ye XH, Yu FH, Dong M. A trade-off between guerrilla and phalanx growth forms in *Leymus secalinus* under different nutrient supplies. Ann Bot –London. 2006; 98: 187–191. 10.1093/aob/mcl086. 10.1093/aob/mcl086 PMC2803537 16687430

[77] Zavaleta ES, Shaw MR, Chiariello NR, Thomas BD, Cleland EE, Field CB (2003). Grassland responses to three years of elevated temperature, CO2, precipitation, and N deposition. Ecol Monog.

[78] Zavaleta ES, Thomas BD, Chiariello NR, Asner GP, Shaw MR, Field CB (2003). Plants reverse warming effect on ecosystem water balance. P Natl Acad Sci USA.

[79] Zhang CY, Li DX (1991). A study on the shoot number changing of *Stipa breviflora* and the ralations with its population yield. Act Agrest Sin.

[80] Zhang YF, Zhang DY (2007). Asexual and sexual reproductive strategies in clonal plants. Front Biol China.

[81] Zhou LY, Zhou XH, He YH, Fu YL, Du ZG, Lu M (2022). Global systematic review with meta-analysis shows that warming effects on terrestrial plant biomass allocation are influenced by precipitation and mycorrhizal association. Nat Commun.

