# Supplementary material for: Warming in combination with increased precipitation mediate the sexual and clonal reproduction in the desert steppe dominant species Stipa breviflora
Source: BMC Plant Biol. 2023 Oct 9;23:474. doi: 10.1186/s12870-023-04439-w (PMC10561481; doi:10.1186/s12870-023-04439-w)
Supplement: Supplementary file 1 — Additional file 1: Figure 1. Air temperature (A) and relative moisture (B) under different warming treatments during the experimental period (from May 28 to August 27, 2017). Figure 2. Effects of warming and increased precipitation on caryopsis biomass (mean ± SE) in Stipa breviflora. Different capital letters represent significant differences among three increased precipitation treatments, and different lowercase letters represent significant differences among three warming treatments at P< 0.05. Figure 3. The Warming and Precipitation Enhancement Platform in Siziwang Station. [file 12870_2023_4439_MOESM1_ESM.docx]

**Warming in combination with increased precipitation mediate the sexual and clonal reproduction in the desert steppe dominant species *Stipa breviflora***

Saixiyala^a, #^, Lingling Chen^a, #^, Fengyan Yi^b^, Xiao Qiu^b^, Hailian Sun^b^, Hongxia Cao^c^, Taogetao Baoyin^a, *^, Xuehua Ye^d, *^, and Zhenying Huang^d^

^a^ Ministry of Education Key Laboratory of Ecology and Resource Use of the Mongolian Plateau & Inner Mongolia Key Laboratory of Grassland Ecology, School of Ecology and Environment, Inner Mongolia University, Hohhot, 010021, China

^b^ Inner Mongolia Academy of Agricultural and Animal Husbandry Sciences, Hohhot, 010030, China

^c^ Suzhou Vocational Technical College, Suzhou, 234099, China

^d^ State Key Laboratory of Vegetation and Environmental Change, Institute of Botany, Chinese Academy of Sciences, Beijing, 100093, China

^#^ Equal first author.

^*^ Corresponding author: Xuehua Ye (yexuehua@ibcas.ac.cn), Taogetao Baoyin (bytgtnm@126.com)

Appendix figures


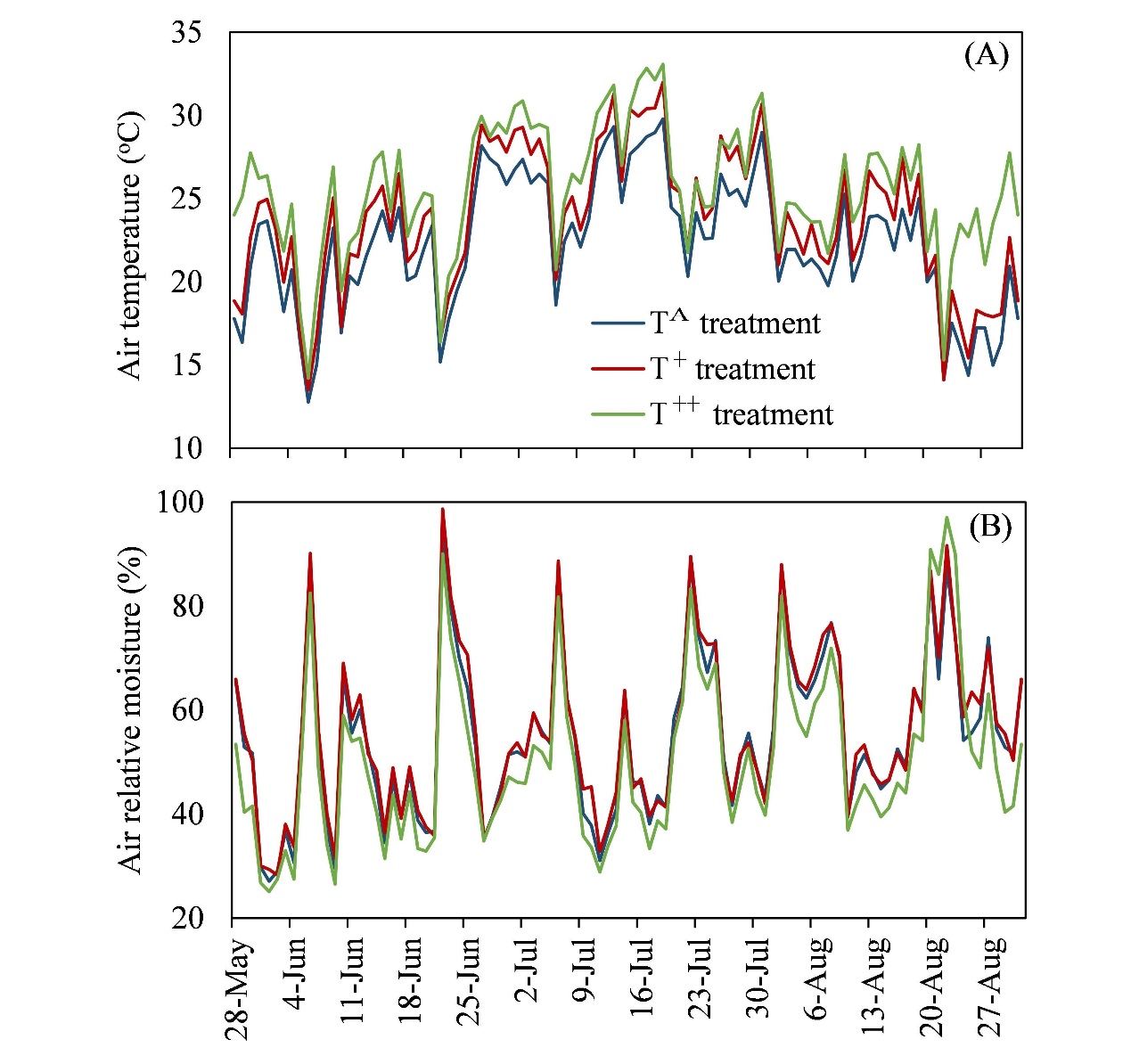


Appendix Figure 1. Air temperature (A) and relative moisture (B) under different warming treatments during the experimental period (from May 28 to August 27, 2017).


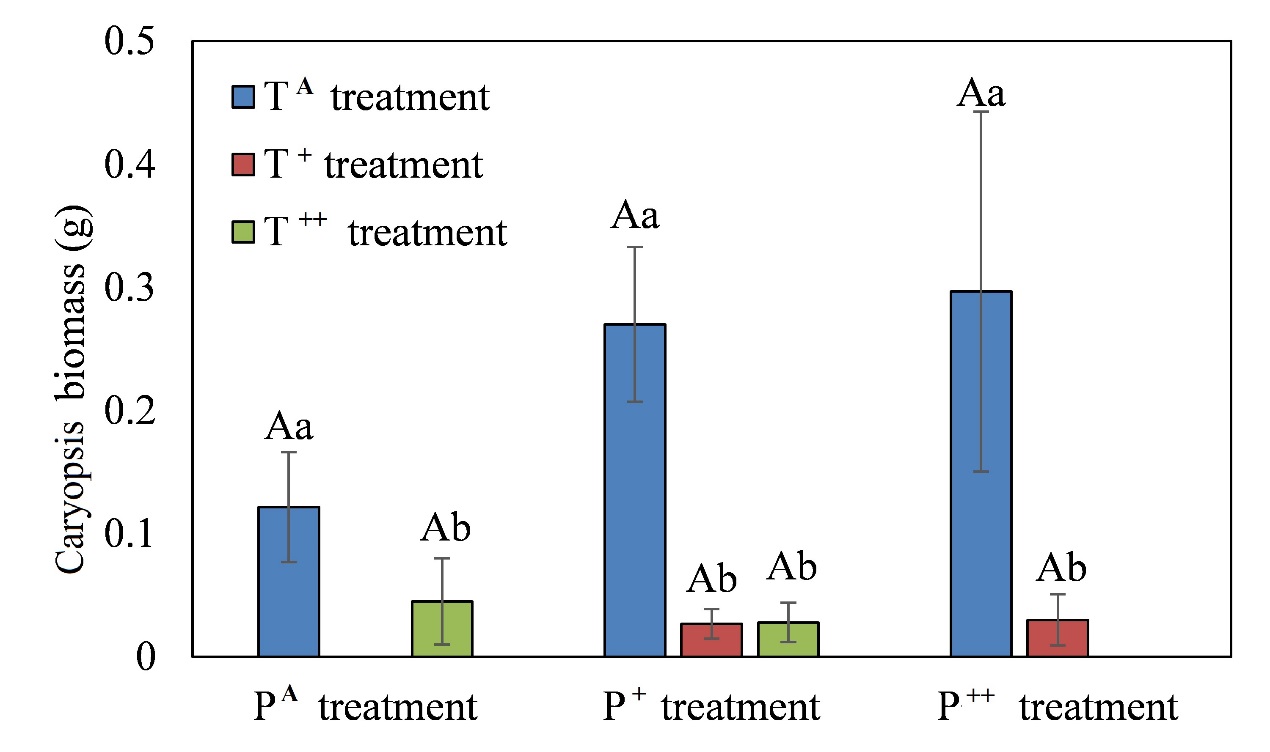


Appendix figure 2. Effects of warming and increased precipitation on caryopsis biomass (mean ± SE) in *Stipa breviflora*. Different capital letters represent significant differences among three increased precipitation treatments, and different lowercase letters represent significant differences among three warming treatments at *P* < 0.05.


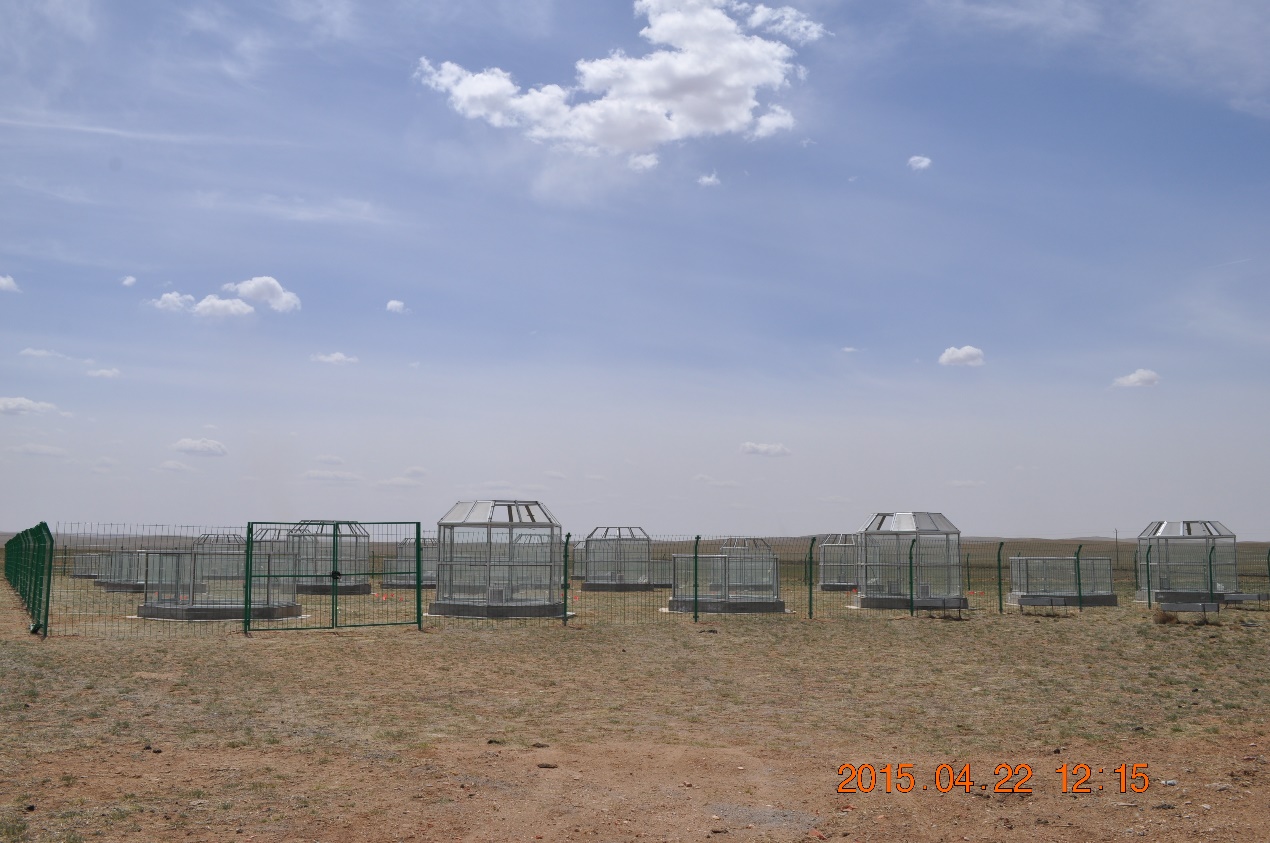


Appendix figure 3. The Warming and Precipitation Enhancement Platform in Siziwang Station.
